# The Effectiveness and Safety of Rhomboid Intercostal Block for Postoperative Pain Management in Thoracic and Breast Surgical Procedures: A Meta-analysis

**DOI:** 10.5812/aapm-150753

**Published:** 2024-10-08

**Authors:** Taufik Saputra, Doso Sutiyono, Widya Istanto Nurcahyo

**Affiliations:** 1 Faculty of Medicine, Diponegoro University, Semarang, Indonesia; 2 Department of Anesthesiology and Intensive Care, Dr. Kariadi General Hospital, Faculty of Medicine, Diponegoro University, Semarang, Indonesia

**Keywords:** Breast Surgery, Opioid Consumption, Pain Score, PONV, Rhomboid Intercostal Block, Thoracic Surgery

## Abstract

**Background:**

The rhomboid intercostal block (RIB) is an emerging regional anesthesia (RA) technique used for pain control following thoracic and breast surgery. However, comprehensive documentation on its effectiveness and safety profile remains limited. This study aims to assess the effectiveness and safety of RIB in thoracic and breast surgical procedures.

**Methods:**

A study search was conducted following PRISMA 2020 guidelines in PubMed, Cochrane Library, Embase, Scopus, and ProQuest from 2016 to 2023 to identify randomized controlled trials (RCTs) evaluating the effectiveness and safety of RIB in thoracic and breast surgeries. The primary outcome was patient pain scores at rest, recorded at one, six, 12, and 24 hours post-surgery. Secondary outcomes included 24-hour opioid consumption and rates of postoperative nausea and vomiting (PONV).

**Results:**

This meta-analysis included five RCTs with a total of 368 patients. Rhomboid intercostal block led to a significant reduction in NRS scores one hour post-surgery (SMD = -1.33; 95% CI = -1.74 to -0.91; P < 0.00001, I² = 18%, P = 0.27), 12 hours post-surgery (SMD = -0.74; 95% CI = -0.99 to -0.48; P < 0.00001, I² = 36%, P = 0.21), and 24 hours post-surgery (SMD = -1.62; 95% CI = -2.56 to -0.69; P = 0.0006, I² = 91%, P < 0.00001). Regarding secondary outcomes, the RIB group showed a significant reduction in 24-hour opioid consumption (SMD = -4.49; 95% CI = -6.09 to -2.90; P < 0.00001, I² = 95%, P < 0.00001) and PONV rates (RR = 0.29; 95% CI = 0.18 to 0.47; P < 0.00001, I² = 0%, P = 0.88).

**Conclusions:**

Rhomboid intercostal block provides effective pain reduction and lowers opioid consumption within 24 hours post-surgery, while also minimizing PONV rates.

## 1. Background

Postoperative pain is one of the most common concerns following thoracic and breast surgical procedures (1, 2). This pain may arise from various mechanisms, including inflammatory, visceral, or somatic sources (3). Over 80% of patients experience acute postoperative pain, with around 75% describing their pain as moderate to extreme. Unrelieved postoperative pain can significantly impact quality of life, hinder functional recovery, and increase the risk of postsurgical complications. Therefore, developing effective strategies for managing postoperative pain is crucial for improving patient outcomes (1, 4).

Opioids and nonsteroidal anti-inflammatory drugs (NSAIDs) are commonly used in pain management strategies, but their side effects can limit their use. Modern approaches to pain management emphasize a multimodal strategy, aiming to reduce opioid reliance, with regional anesthesia (RA) playing a central role (3, 5). Various RA techniques, such as intercostal nerve blocks, paravertebral blocks, and thoracic epidural anesthesia, are utilized to reduce postoperative pain. However, the analgesic effects of intercostal nerve blocks are short-lived, and paravertebral blocks and thoracic epidurals can lead to parasympathetic symptoms such as hypotension and bradycardia (6).

**Figure 1.**
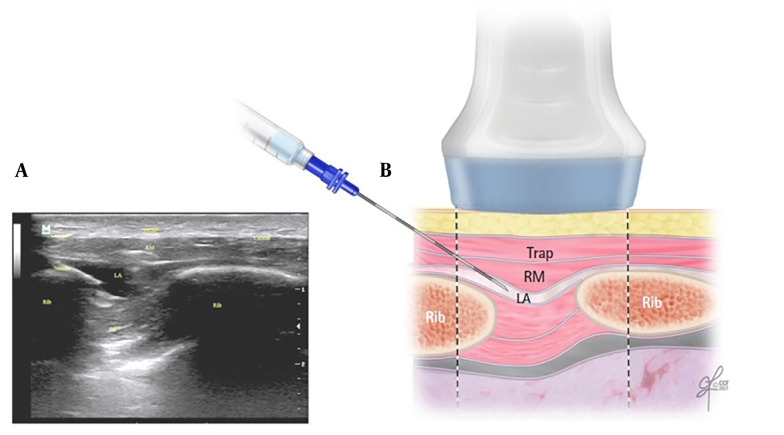
A, the corresponding ultrasound image; B, schematic illustration showing the surrounding structures and needle position for performing the rhomboid intercostal injection at the T5 and T6 levels. IM, intercostal muscles; LA, local anesthetic; RM, rhomboid major muscle; Trap, trapezius muscle. Reproduced from Elsharkawy H, Hamadnalla H, Altinpulluk EY, Gabriel RA. Rhomboid intercostal and subserratus plane block -a case series. Korean J Anesthesiol. 2020 Dec 1;73(6):550-6 (9).

In 2016, Elsharkawy et al. introduced a novel RA technique known as the rhomboid intercostal block (RIB) (7). Rhomboid intercostal block involves injecting a local anesthetic into the upper intercostal muscle plane beneath the rhomboid muscles, providing analgesia to both the anterior and posterior thorax (Figure 1) (8). In recent years, several randomized controlled trials (RCTs) have examined the efficacy of RIB in thoracic and breast surgeries. Despite this, comprehensive documentation regarding the postoperative pain outcomes and safety profile of this technique remains limited.

## 2. Objectives

To address this gap, we conducted a systematic review and meta-analysis to thoroughly evaluate the efficacy and safety of RIB.

## 3. Methods

Under the guidelines of the preferred reporting items for systematic reviews and meta-analyses (PRISMA) statement, this study evaluated the effectiveness and safety of the RIB for analgesia in thoracic and breast surgical procedures (10).

### 3.1. Search Strategy

A comprehensive systematic literature search was conducted using PubMed, Cochrane Library, Embase, Scopus, and ProQuest from 2016 to 2023. The search, performed until October 30, 2023, utilized primary keywords and related terms such as “rhomboid intercostal block,” “rhomboid intercostal nerve block,” “thoracic surgery,” and “breast surgery” in various combinations. These were selected according to the PICO framework, detailed as follows:

P (population): Patients undergoing thoracic or breast surgery; I (intervention): Rhomboid intercostal block for thoracic or breast surgery; C (control): No block or placebo; O (outcomes); primary: Comparison of Numerical Rating Scale (NRS) scores; secondary: Comparison of 24-hour opioid consumption and postoperative nausea and vomiting (PONV)

The inclusion criteria were as follows: (1) RCTs; (2) population of 15 years old and older; (3) patients undergoing thoracic or breast surgery; (4) an experimental group treated with a single-shot RIB and a control group with no block; and (5) full-text publications of human studies written in English.

The exclusion criteria were: (1) non-RCTs, such as reviews, case reports, animal experiments, and in vitro studies; (2) studies where RIB was not mentioned; and (3) studies with no control group.

### 3.2. Study Extraction

The titles and abstracts of all publications identified through the search were independently reviewed by TS (the primary reviewer) and DS (the secondary reviewer). Full-text copies of all studies deemed potentially eligible were obtained. After reviewing the full texts, the reviewers reassessed the studies and applied the eligibility criteria to exclude additional papers. Any disagreements were resolved through repeated discussions until consensus was reached. In cases where the two reviewers could not reach an agreement, a third reviewer (WIN) provided the final decision. Once a final consensus was achieved, the data extraction sheet was completed. The extraction form included details such as the first author, year of publication, study design, type of surgery, number of patients enrolled in each surgery type, intervention performed, and reported outcomes.

### 3.3. Risk of Bias Assessment

All studies included in this systematic review and meta-analysis were evaluated for risk of bias based on their study design. Two investigators (TS, DS) independently assessed each study, using the updated cochrane risk of bias tool for randomized trials (RoB 2.0) (11). In cases where different scores were assigned, any discrepancies were resolved through discussion until consensus was reached.

### 3.4. Quality of Evidence

The grades of recommendation, assessment, development, and evaluation (GRADE) guidelines were applied to assess the quality of evidence for each individual outcome (12). Grades of recommendation, assessment, development, and evaluation evaluates five key categories—risk of bias, imprecision, inconsistency, indirectness, and publication bias—to determine factors that may impact the quality of evidence. Based on these factors, the quality of evidence for each outcome was classified into one of four levels: high, moderate, low, or very low.

### 3.5. Primary and Secondary Outcomes

Postoperative NRS scores at rest, recorded at one, six, 12, and 24 hours after surgery, served as the primary outcomes. Secondary outcomes included 24-hour postoperative opioid consumption and the incidence of PONV.

### 3.6. Statistical Analysis

The data extracted by the two investigators was cross-checked for accuracy. Review Manager version 5.4 (The Nordic Cochrane Centre, The Cochrane Collaboration, Copenhagen, Denmark) was used to analyze the extracted data. For dichotomous data, relative risk (RR) and 95% confidence intervals (CIs) were calculated, while continuous variables were assessed using standardized mean difference (SMD) and 95% CIs. The Cochrane I² statistic was employed to assess statistical heterogeneity. Random effect models were applied in cases of significant heterogeneity (I² > 50%); otherwise, fixed effect models were used (13, 14). Statistical significance for all outcomes was set at P < 0.05 with 95% CIs.

## 4. Results

**Figure 2.**
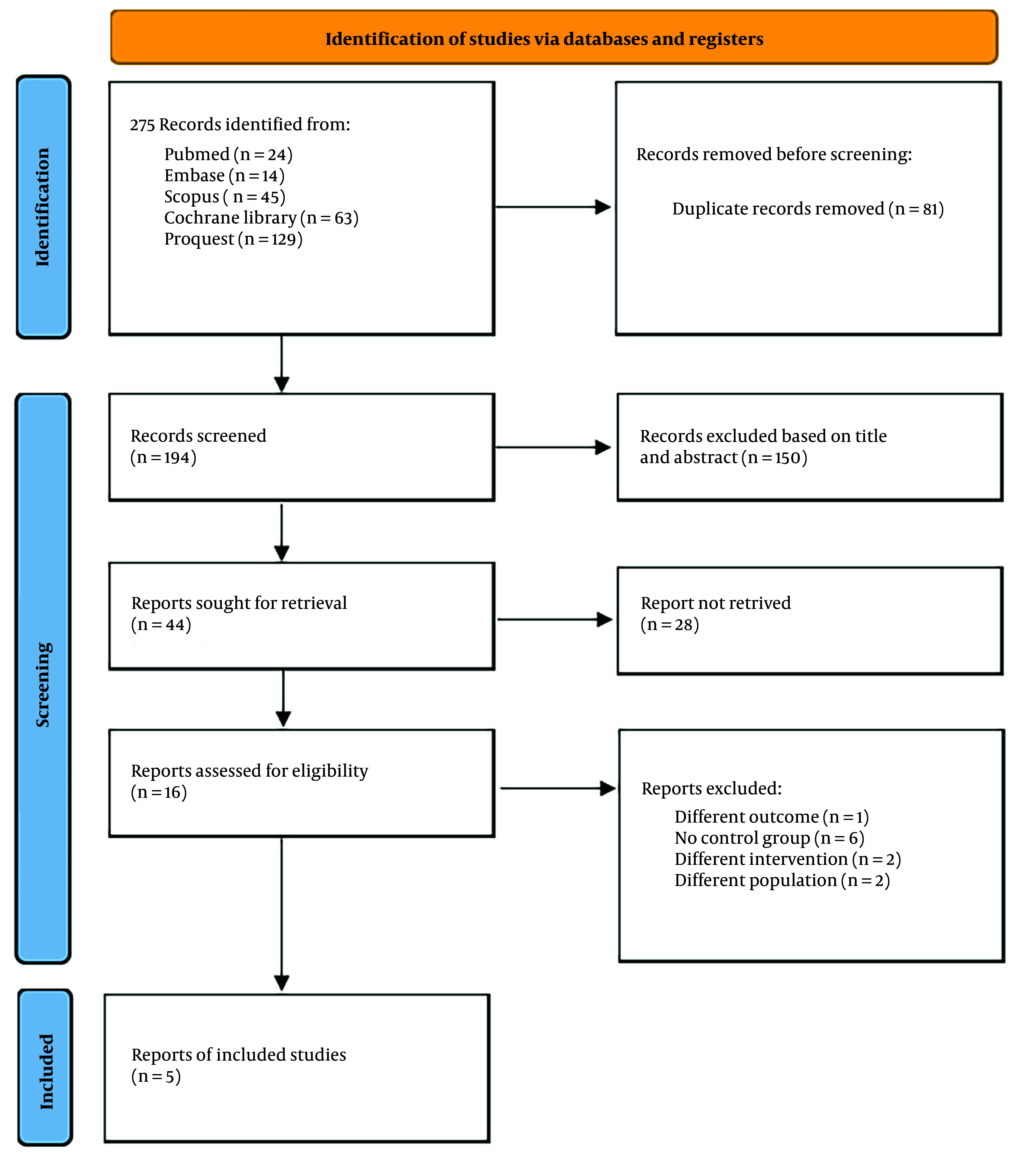
PRISMA Flow diagram of included and excluded studies

After conducting a database search that initially generated 275 records, we reviewed the summaries of those records to assess eligibility. Following a filtering process to remove duplicates, 194 unique articles remained. After title and abstract screening, 16 studies were selected for full-text review. Further screening led to the exclusion of 11 studies for various reasons, leaving five eligible studies from databases and registers. These five trials included a total of 368 patients, with 184 assigned to the RIB group and 184 in the control group without blocks (6, 15-18) Figure 2 presents a flow diagram illustrating the research strategy and selection procedure.

**Figure 3.**
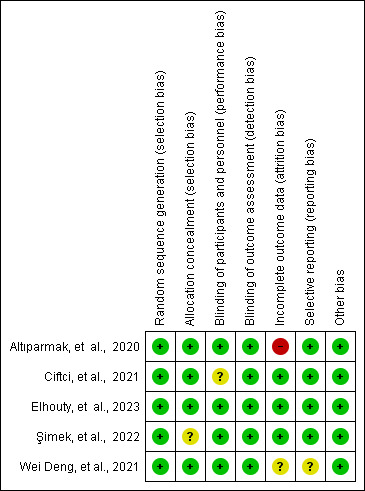
Risk of bias 2.0. of randomized controlled trials (RCTs). Green circle, low risk of bias; yellow circle, unclear risk of bias; red circle, high risk of bias.

All five studies (6, 15-18) were RCTs published in peer-reviewed journals. One trial compared RIB to no block, while another compared RIB to both no block and type-II pectoral nerve block (PECS). A third trial compared RIB with no block and the serratus anterior plane block (SAPB), while a fourth compared RIB with no block and the erector spinae plane block. The final trial compared RIB with no block and the rhomboid intercostal and subserratus plane block (RISS). Each of these comparisons was analyzed independently. Table 1 provides a summary of the specific features of the included studies and the GRADE results are summarized in Table 2. A risk of bias assessment using RoB 2.0 showed that all studies had a low risk of bias (Figure 3). 

**Table 1.** Studies Characteristic

Authors	Year	Study Design	Country	Number of Patients	Mean Ages	Type of Surgery	RIB Dose	Opioid
**Elhouty et al. (** 17 **)**	2023	RCT	Egypt	142: RIB (71); control (71)	RIB: 23.2 ± 4.25; control: 23.7 ± 4.1	VATS	20 mL 0.25% bupivacaine	Fentanyl
**Şimek et al. (** 18 **)**	2022	RCT	Turkey	50: RIB (25); control (25)	RIB: 60.2 ± 10.2; control: 54.8 ± 12.2	Elective resection of non-metastatic lung malignancies	20 mL 0.25% bupivacaine	Tramadol
**Ciftci et al. (** 16 **)**	2021	RCT	Turkey	60: RIB (30); control (30)	RIB: 49.4 ± 3.7; control: 43.1 ± 4.2	Unilateral BCS-AD and axillary dissection surgery	30 mL 0.25% bupivacaine	Fentanyl
**Deng et al. (** 6 **)**	2021	RCT	China	60: RIB (30); control (30)	RIB: 60.5 ± 11.6; control: 56.6 ± 11.5	VATS	20 mL 0.375% ropivacaine	Sufentanil
**Altipamark et al. (** 15 **)**	2020	RCT	Turkey	56: RIB (28); control (28)	RIB: 53.8 ± 11.2; control: 52.0 ± 11.5	Unilateral Modified Radical Mastectomy	30mL 0.25% bupivacaine	Morfin

Abbreviation: RIB, rhomboid intercostal block.

**Table 2.** Certainty Assessment Using Grades of Recommendation, Assessment, Development, and Evaluation Approach

Outcomes	No. of Participants (Studies)	Certainty of the Evidence (GRADE)	Comments
**1 h NRS score**	308 (4 RCTs)	⨁⨁⨁⨁ (high)	
**6 h NRS score**	308 (4 RCTs)	⨁⨁⨁◯ (moderate)	Inconsistency" was downgraded to "serious."
**12 h NRS score**	252 (3 RCTs)	⨁⨁⨁⨁ (high)	
**24 h NRS score**	368 (5 RCTs)	⨁⨁⨁◯ (moderate)	Inconsistency" was downgraded to "serious."
**24 h opoid consumption**	368 (5 RCTs)	⨁⨁⨁◯ (moderate)	Inconsistency" was downgraded to "serious."
**PONV**	226 (4 RCTs)	⨁⨁⨁⨁ (high)	

Abbreviations: GRADE, grades of recommendation, assessment, development, and evaluation; RCTs, randomized controlled trials.

### 4.1. Primary Outcome

For the assessment of postoperative pain, two studies utilized NRS scores (6, 15), while three studies used VAS scores (16-18). Since NRS and VAS scores are comparable, the VAS scores were converted to NRS values for consistency in comparison (19).

### 4.2. Numerical Rating Scale Scores at Rest Recorded 1 h Post-surgery

**Figure 4.**
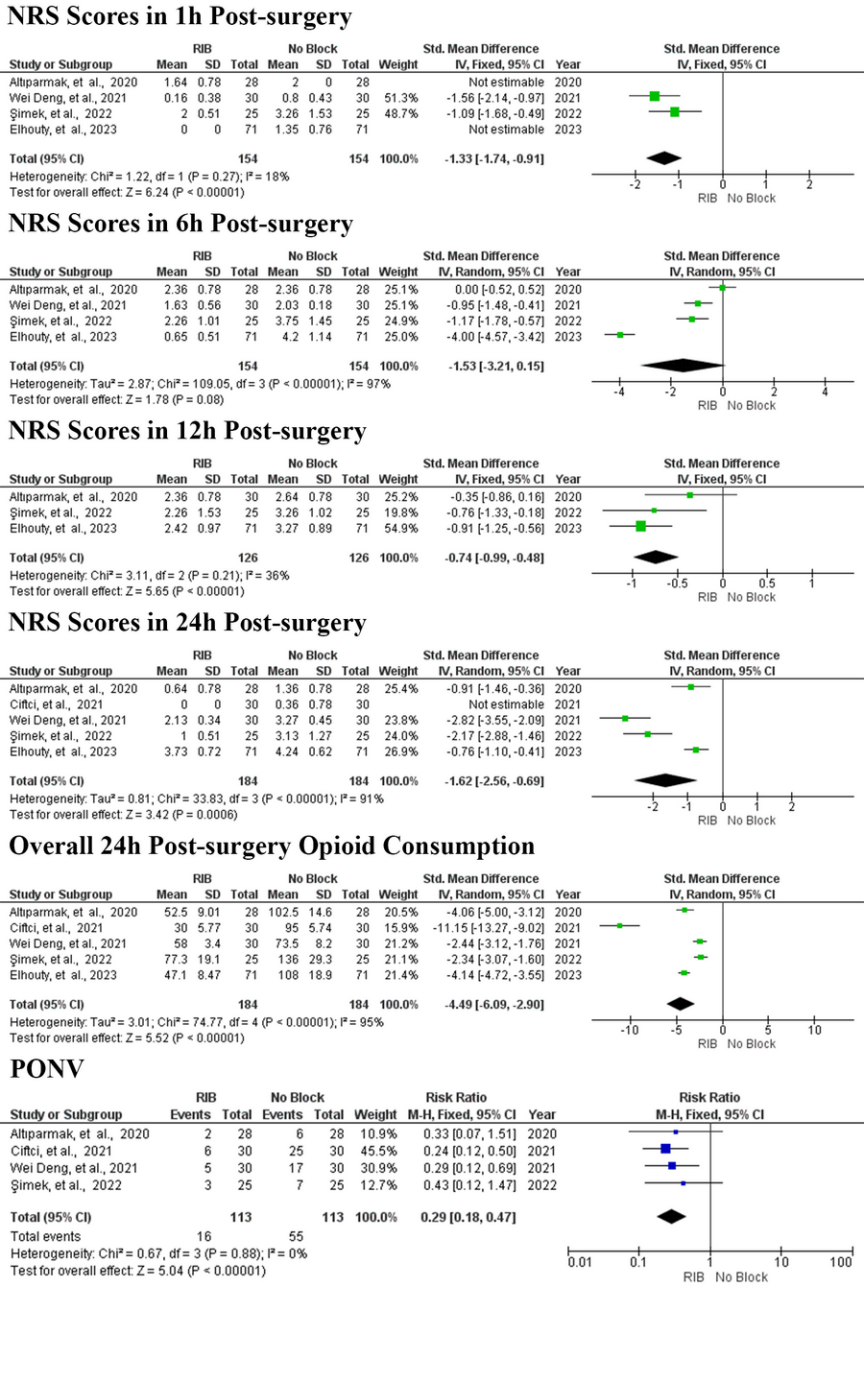
Forest plot of the primary and secondary outcomes. Squares show the SMD and OR estimates for each study, and the lines that cross them show the 95% confidence interval. The overall pooled estimate is shown by the diamond. SMD, standardized mean difference; RR, relative risk; CI, confidence interval; IV, inverse variance; M–H, mantel Haenszel; SD, standard deviation; RIB, rhomboid intercostal block.

A forest plot was created to compare NRS scores at rest one hour post-surgery for patients receiving RIB versus no block, based on the results of four studies (Figure 4) (6, 15, 17, 18). The analysis utilized a fixed-effects model. As shown in Figure 3, the total number of patients across these four studies was 308, with 154 patients in the RIB group and 154 in the control group. The results indicated that RIB significantly reduced NRS scores at rest one hour post-surgery compared to the no block group (SMD = -1.33; 95% CI = -1.74 to -0.91; P < 0.00001, I^2^ = 18%, P = 0.27).

### 4.3. Numerical Rating Scale Scores at Rest Recorded 6h Post-surgery

The forest plot (Figure 4) illustrates the comparison of NRS scores at rest six hours post-surgery between RIB and no block. This analysis, based on data from four studies (6, 15, 17, 18) and employing a random-effects model, revealed considerable heterogeneity across trials, even after conducting sensitivity analyses that excluded individual studies one by one. The total number of patients in these four studies was 308, with 154 in the RIB group and 154 in the control group. The results indicated that RIB did not lead to a statistically significant reduction in NRS scores at rest six hours post-surgery compared to the no block group (SMD = -1.53; 95% CI = -3.21 to 0.15; P = 0.08, I^2^ = 97%, P < 0.00001).

### 4.4. Numerical Rating Scale Scores at Rest Recorded 12h Post-surgery

The forest plot (Figure 4) displays the comparison of NRS scores at rest 12 hours post-surgery for RIB versus no block based on three studies (15, 17, 18).A fixed-effects model was applied for this analysis, encompassing a total of 252 patients—126 in the RIB group and 126 in the control group. The findings showed a significant reduction in NRS scores at rest at 12 hours post-surgery for the RIB group compared to the no block group (SMD = -0.74; 95% CI = -0.99 to -0.48; P < 0.00001, I^2^ = 36%, P = 0.21).

### 4.5. Numerical Rating Scale Scores at Rest Recorded 24h Post-surgery

The forest plot (Figure 4) illustrates the comparison of NRS scores at rest 24 hours post-surgery for RIB versus no block, based on five studies (6, 15-18). A random-effects model was applied, revealing significant heterogeneity between trials despite sensitivity analyses that omitted individual studies alternately. The total number of patients included across these five studies is 368, with 184 patients in both the RIB and control groups. The analysis demonstrated a significant reduction in NRS scores at rest 24 hours post-surgery for the RIB group compared to the no block group (SMD = -1.62; 95% CI = -2.56 to -0.69; P = 0.0006, I^2^ = 91%, P < 0.00001).

### 4.6. Secondary Outcomes

#### 4.6.1. Overall 24 h Post-surgery Opioid Consumption

In terms of postoperative opioid consumption, two studies utilized fentanyl (16, 17), one used morphine (15), one used tramadol (18), and one used sufentanil (6). To standardize the data, the morphine, tramadol, and sufentanil doses were converted to fentanyl equivalents (20). However, the study using sufentanil was excluded from the analysis as its converted fentanyl dose was significantly lower than in the other groups, which could skew the results.

The forest plot (Figure 4) presents opioid consumption within 24 hours post-surgery. A random-effects model was applied to calculate the overall opioid consumption, and sensitivity analysis showed that even after omitting individual studies alternately, significant heterogeneity persisted between trials. Across the four remaining studies, 368 patients were included, with 184 in both the RIB and control groups. The analysis revealed that RIB significantly reduced opioid consumption within 24 hours post-surgery compared to the no block group (SMD = -4.49; 95% CI = -6.09 to -2.90; P < 0.00001, I^2^ = 95%, P < 0.00001).

#### 4.6.2. Postoperative Nausea and Vomitting 

In four studies, the rates of PONV related to opioid use were assessed (6, 15, 16, 18). The results are depicted in the forest plot (Figure 4). A fixed-effects model was used for the analysis. The total number of patients across these four studies was 226, with 113 in the RIB group and 113 in the control group. The analysis demonstrated that RIB significantly reduced PONV rates compared to the no block group (RR = 0.29; 95% CI = 0.18 to 0.47; P < 0.00001, I^2^ = 0%, P = 0.88).

## 5. Discussion

This meta-analysis, encompassing five RCTs with a total of 368 patients, evaluated the analgesic effectiveness and safety of RIB in patients undergoing thoracic and breast surgical procedures. Compared to the control group, RIB significantly reduced pain scores at rest at various time points and markedly decreased opioid consumption during the first 24 hours post-surgery. Additionally, RIB was associated with a lower incidence of PONV.

While thoracic epidural analgesia (TEA) has long been considered the gold standard for RA following thoracic and breast surgeries, it comes with certain risks (21, 22). These include complications such as spinal abscess, dural puncture, epidural hematoma, and significant hemodynamic effects due to sympathetic blockade from local anesthesia (18).

Rhomboid intercostal block is a novel interfascial plane block administered in the area along the medial border of the scapula, known as the triangle of auscultation (7, 23). Previous cadaveric investigations using methylene blue contrast have shown that the dye spreads between the rhomboid major and intercostal muscles from the T2 to T8 levels, both cranially and caudally. Additionally, staining was observed in the posterior rami of the thoracic spinal nerves at T2-T9 levels and the lateral cutaneous branches of the intercostal nerves from T2 to T8 (7, 18) This suggests that RIB can provide effective analgesia for both the anterior and posterior hemithorax, making it suitable for thoracic and breast surgical procedures.

The RIB group significantly reduced pain levels at rest compared to the no block group at one, 12, and 24 hours post-surgery, based on NRS scores. Additionally, the RCTs included in this meta-analysis compare the RIB group with other block techniques. RIB was shown to be superior to the serratus anterior plane block (SAPB) due to its association with better analgesic outcomes (17). However, other studies found that RIB provided similarly effective analgesia when compared to the erector spinae plane block (ESPB) and type-II pectoral nerve block (PECS II) (16, 18). While neither PECS II nor RIB is superior to the other, the PECS II block has the disadvantage of having puncture points near the surgical site (15).

In terms of 24-hour postoperative opioid consumption, the RIB group consumed significantly fewer opioids than the no block group. This reduction in opioid use may offer potential benefits by decreasing opioid-related adverse effects, such as bronchoconstriction at high doses, cough suppression, chest wall rigidity, and dose-dependent respiratory depression (24). Additionally, lower opioid doses can reduce the risk of opioid-induced hyperalgesia (OIH), a condition where patients using opioids for pain management become more sensitive to painful stimuli, leading to poorly controlled pain and the need for higher doses (25, 26). In summary, reduced opioid consumption may contribute to improved recovery during the postoperative period.

In terms of one of the most common opioid-related complications, namely PONV, the RIB group showed an incidence of 14.16%, whereas the no block group had a significantly higher incidence of 48.67%. Opioids can induce PONV by directly affecting receptors in the brainstem's chemoreceptor trigger zone (27). Postoperative opioid use typically increases the risk of PONV in a dose-dependent manner, with the effect persisting as long as opioids are administered (28). Although PONV may be transient or mild, its impact on patients can be severe, leading to delayed recovery, difficulties with mobilization, and reduced oral intake (29). The lower incidence of PONV in the RIB group may be attributed to the minimal opioid consumption within 24 hours post-surgery. None of the studies in this meta-analysis reported any block-related complications. Therefore, it can be concluded that RIB is a relatively safe blocking technique.

It is important to acknowledge several limitations of this meta-analysis. First, despite using a random-effects model and applying strict inclusion and exclusion criteria to standardize the selected studies, there remains significant heterogeneity in the results. Sensitivity analyses demonstrated that even when individual studies were alternately omitted, a considerable amount of heterogeneity persisted among the RCTs. The primary causes of this heterogeneity may include variations in injection levels, agent concentrations, and volumes. However, conducting a meta-regression to assess the impact of these potential variables was not feasible due to the limited number of available studies.

Second, the studies included in this analysis had relatively small sample sizes, which may limit the strength of the conclusions. Larger, multicenter RCTs are needed to further investigate and solidify the findings in this area. Finally, there is always some uncertainty when pooling data for a meta-analysis, particularly when transforming median and range values into mean and SD values, which can introduce additional variability into the effect size estimates.

In conclusion, this meta-analysis demonstrated that the RIB group experienced significantly lower NRS scores, reduced 24-hour opioid consumption, and lower rates of PONV compared to the no block group. Future RCTs with more standardized reporting are essential to validate and expand upon the findings of previous studies and this meta-analysis.

## Data Availability

The data presented in this study are uploaded during submission as a supplementary file and are openly available for readers upon request.
